# Lead Discovery Strategies for Identification of *Chlamydia pneumoniae* Inhibitors

**DOI:** 10.3390/microorganisms4040043

**Published:** 2016-11-28

**Authors:** Leena Hanski, Pia Vuorela

**Affiliations:** Pharmaceutical Biology, Division of Pharmaceutical Biosciences, Faculty of Pharmacy, University of Helsinki, Helsinki FI-00014, Finland; pia.vuorela@helsinki.fi

**Keywords:** *Chlamydia pneumoniae*, antichlamydial agent, plant phenolics, antimicrobial peptide

## Abstract

Throughout its known history, the gram-negative bacterium *Chlamydia pneumoniae* has remained a challenging target for antibacterial chemotherapy and drug discovery. Owing to its well-known propensity for persistence and recent reports on antimicrobial resistence within closely related species, new approaches for targeting this ubiquitous human pathogen are urgently needed. In this review, we describe the strategies that have been successfully applied for the identification of nonconventional antichlamydial agents, including target-based and ligand-based virtual screening, ethnopharmacological approach and pharmacophore-based design of antimicrobial peptide-mimicking compounds. Among the antichlamydial agents identified via these strategies, most translational work has been carried out with plant phenolics. Thus, currently available data on their properties as antichlamydial agents are described, highlighting their potential mechanisms of action. In this context, the role of mitogen-activated protein kinase activation in the intracellular growth and survival of *C*. *pneumoniae* is discussed. Owing to the complex and often complementary pathways applied by *C. pneumoniae* in the different stages of its life cycle, multitargeted therapy approaches are expected to provide better tools for antichlamydial therapy than agents with a single molecular target.

## 1. Introduction

Since its identification as a district species in 1980s [1,2], *Chlamydia pneumoniae* has been recognized as an ubiquitous human pathogen, responsible for 5%–10% of community-acquired pneumonia cases and causing a spectrum of respiratory tract infections with varying severity. Based on serological studies, this bacterium is currently known to be present in the populations of both developed and developing countries and to infect the majority of people at least once in a lifetime. Within the 30 years of research on *C. pneumoniae*, this small spherical bacterium has presented itself as a challenging target for antimicrobial therapy and drug development. Inherent challenges in the antichlamydial therapy link to the Gram-negative yet morphologically unique cell wall of Chlamydiaceae and the obligate intracellular, intravacuolar replication of this family of bacteria. The chlamydial outer membrane consisting of family-specific lipooligosaccharides creates a permeability barrier that typically limits the entry of antibiotics into gram-negative bacteria. The inclusion membrane surrounding *C*. *pneumoniae* during its intracellular stages forms an additional barrier towards antibiotics; in contrast to many other vacuolar parasites, the Chlamydiaceae-associated inclusion membrane does not allow entry of components with an approximate size of greater than 500 Da by passive diffusion [3]. A relatively small size, sufficient tissue penetration and ability to pass through eukaryotic cell membranes are therefore necessary for any therapeutic agent targeting structures of replicating *Chlamydia* spp. bacteria.

In clinical settings, *C*. *pneumoniae* infections are associated with relapsing symptoms and treatment failures even when the first-choice antibiotics are used. Up to 30% of patients with *C. pneumoniae*-caused community acquired pneumonia have been reported to harbor the bacterium in a cultivable form even after the treatment and symptoms have ceased [4,5]. According to current knowledge, chlamydial persistence, rather than resistance, is responsible for most treatment failures. To date, no resistant mutants of *C*. *pneumoniae* have been isolated from clinical samples and even the strains originating from patients with treatment failure do not show altered in vitro susceptibility profiles to antibiotics. Documentation on tetracycline resistance in a related porcine pathogen *Chlamydia suis* and reports on the transfer of genetic material between chlamydial species, recently reviewed by Borel et al. [6], demonstrate that the possibility of homotypic and heterotypic resistance must also be considered in the future in human chlamydial infections.

In our previous review article, we have discussed the nonconventional antichlamydial agents identified within past ten years and highlighted the need for more narrow-spectrum or even chlamydia-specific antibacterial agents [7]. In the current contribution, we focus on describing the different approaches for antichlamydial drug discovery, with illustrative examples of successful use of each of them. In addition, some recent advances in the characterization and translational work on the nonconventional antichlamydial compounds are presented. Regarding the mechanisms of action, special emphasis is put on discussing the various cellular processes affected by both *C*. *pneumoniae* and a potent antichlamydial agent luteolin.

## 2. Lead Discovery Strategies

Besides the above mentioned inherent challenges in antichlamydial therapies, discovery of new antichlamydial agents faces some major technical limitations due to the long developmental cycle and genetic intractability of *C*. *pneumoniae*. These features make large-scale screens of small molecule libraries against *C*. *pneumoniae* unattractive, slow and technically demanding. Here, we describe more focused and hypothesis-driven approaches that have proven useful in the attempts to find new inhibitors of *C. pneumoniae*.

### 2.1. Epidemiological/Ethnopharmacological Approach

Epidemiological and ethnopharmacological approaches as the basis for finding novel bioactive compounds stem from the observations on health-promoting effects of dietary components and herbal remedies. As a result of long coevolution with other species, secondary metabolites of plants and other organisms have proven to be a fruitful source of small molecules interacting and interfering with animal and microbial cells. This is well illustrated by the fact that approximately half of currently approved drugs are of natural origin, and biogenic drugs are especially abundant in the antimicrobial therapy areas [8].

Connection of *C. pneumoniae* infection to atherosclerosis and other inflammatory diseases, along with the well-known protective role of dietary polyphenols in these diseases, have triggered the question on a potential link between *C. pneumoniae* and the phenolic compounds. Inspired by the French paradigm, red wine extract has been shown to suppress *C. pneumoniae* growth [9]. In our previous screening study of 93 Finnish plant extracts for their inhibitory effects on *C*. *pneumonia,* several plant extracts, including blueberry, thyme and different mint species, were found to suppress *C. pneumoniae* growth in an epithelial cell line [10]. Based on its high antichlamydial activity and traditional use for treating respiratory tract symptoms, corn mint (*Mentha arvensis* L.) was selected for further characterization. The antichlamydial effect of corn mint was characterized in a mouse model of acute *C*. *pneumoniae* infection, revealing a decrease in the number of *C. pneumoniae*-positive animals and *C. pneumoniae*-specific IgG antibodies among the extract-treated mice. Bioactivity-guided fractionation revealed two phenolic compounds, linarin and rosmarinic acid as the main antichlamydial components of the extract [11].

The studies on polyphenol-rich plant extracts prompted the systematic investigation of plant phenolics in purified form as antichlamydial agents. In a study of 57 compounds selected to cover the typical classes of phenolic compounds, gallates, flavones and flavonoles were identified as the most active types of the studied phenolics against *C*. *pneumoniae* clinical isolate K7 in epithelial cells [12].

The antichlamydial activity of luteolin, a flavonol present in apples, broccholi, parsley and several other dietary plants, has also been studied in vivo using the mouse model on acute *C. pneumoniae* infection, revealing similar results in a relative number of *C. pneumoniae*-positive animals and *C. pneumoniae*-specific IgG titer as described above on the corn mint extract [13]. Luteolin and various other dietary phenolics have been extensively studied on their cellular and molecular level activities, and besides guiding our everyday dietary choices, the ultimate value of the identification of the antichlamydial activities of these compounds can be seen in the insights into the intracellular survival mechanisms that might be reached via further mechanistic studies. With this in mind, the latter part of this review describes some of the cellular processes that are potentially involved in the interactions between dietary phenolics and *C. pneumoniae*.

The potential of further mining the traditionally used medicinal plants for antichlamydial compounds is also supported by recent findings with a reverse approach. Using a high-content screening assay for the identification of antichlamydial compounds in a diversity-based natural product library, schisandrin B was found to suppress *C*. *pneumoniae* growth [14]. This dibenzocyclooctadiene lignin, together with its close structural analogues, is the dominant chemical components in the fruit extract of *Schisandra chinensis* (Turcz. Baill.) commonly known as wu wei zi or five-flavour berry, an extensively applied adaptogenic and liver-protecting medication in eastern medicine. Among its many other uses, the *S*. *chinensis* fruit extract has been used to alleviate respiratory tract symptoms, especially prolonged coughing [15]. The antichlamydial activity of the extract has also been demonstrated [16], and even though the original findings were not driven by the ethnopharmacological hypothesis, the traditional indication of the extract fits well with the typical clinical manifestation of *C. pneumoniae* infections.

### 2.2. Target-Based Virtual Screening

Challenged by the fact that existing antibacterial drugs target only a handful of bacterial molecules, new antibiotics with novel mechanisms of action are urgently needed. Relying on the concept of the essential role of individual proteins in a given disease, target-based drug discovery has been at the core of rational drug discovery for several decades. The inherent prerequisite for effective target-based discovery is the well-validated nature of the target protein, which in the case of antimicrobial drug discovery, reflects the indispensable role if the molecule for either microbial growth or virulence.

In the context of *C*. *pneumoniae* infections, this process is hindered by the lack of data on suitable chlamydia-specific proteins that could be targeted to block bacterial replication or block its virulence mechanisms. One of the few chlamydial targets with evidence on the remarkable, indispensable role in the intracellular survival of *C. pneumoniae* is chlamydial protease-like activity factor (CPAF). This chlamydial effector protein is known to be secreted from the infection vacuole once effective replication has been established and, as its name indicates, cleave host cell proteins for the chlamydial favour [17]. In 2011, Christian et al. reported on findings demonstrating the ability of *C. pneumoniae* CPAF to induce Golgi fragmentation [18]. This feature was reported to be vital for *C*. *pneumoniae* survival, allowing the authors to conclude that targeting CPAF could be an effective strategy to suppress chlamydial infections. In addition, CPAF has been shown to disrupt host cell proinflammatory signaling by cleaving a NF-κB family transcription factor p65, indicating the role of CPAF as a chlamydial virulence factor by limiting host cell sensitivity to proinflammatory stimuli [19]. CPAF crystal structure has been resolved as a complex with a protease inhibitor lactocystin, and based on a proteomic analysis host epithelial cells upon *C*. *pneumoniae* infection, a target recognition sequence for CPAF has been suggested [20]. These data could provide starting points for rational design of CPAF inhibitors, but the effectiveness of any compounds identified on CPAF can be expected to cover only acute chlamydial infections. Even though the role of CPAF for successful acute infection seems indispensable, the translocation of CPAF to host cell cytoplasm is inhibited in persistent infection [17], which suggests that targeting CPAF may not represent an effective strategy for eradication of persistent infections.

Besides the lack of validated targets, target-based drug discovery on *C*. *pneumoniae* is limited by the fact that structural studies on *C*. *pneumoniae* proteins have focused on immunogenic surface proteins. Thus, very limited number of 3D structures of other *C. pneumoniae* proteins that could be used as the basis for docking screens of virtual small molecule libraries is available. To overcome these limitations, a similarity-based screen of *C*. *pneumoniae* genes in other organisms, combined with homologue modeling of the protein structure, can be applied.

**Figure 1:**
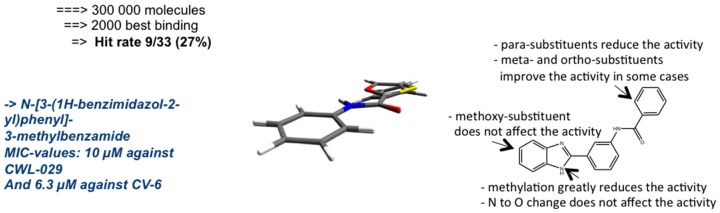
The lead optimization gives information via structure–activity relationships that will be basis to synthesize the next generation of lead compounds.

Following this approach, a search for *C*. *pneumoniae* genes encoding proteins with no human analogues but showing high similarity to other bacterial genes associated with known protein 3D structures identified *C*. *pneumoniae* dimethyladenosine transferase (coded by the *ksgA* gene) as a close homologue to a *Bacillus subtilis ermC* gene, encoding a RNA methyltransferase [21]. Both genes encode for enzymes belonging to RNA (adenine-N6-)-methyltransferases (EC 2.1.1.48), mediating essential functions in ribosomal methylation and structure. The *B. subtilis* ermC crystal structure was used for a virtual screen of 300,000 compounds (Figure 1), followed by growth inhibition assays on *C. pneumoniae*, with 33 compounds selected to represent the most probable ligands of the enzyme active site. Among the 33 assayed compounds, eight previously unknown antichlamydial compounds were identified. Further lead optimization has been carried out on compounds with a benzimidazole core structure by the design and synthesis of new derivatives bearing this skeleton as well as structure-activity relationship studies, demonstrating several derivatives with MIC values in low micromolar range and indicating the favourable impact of non-planar conformation of the 2-arylbenzimidazole structure for the antichlamydial activity [22,23].

Other studies on these benzimidazole derivatives have shown that they do not exert broad-spectrum antibacterial activities, and despite their in silico affinity to *ermC* active site, their additional or alternative molecular targets are yet to be identified in vitro.

### 2.3. Ligand-Based Virtual Screening

Despite the established role of target-based approaches in drug discovery over the past few decades, this strategy has generally failed to produce new antibacterial drug candidates [24]. Massive efforts by the pharmaceutical industry have been made to evaluate the 100–200 conserved bacteria-specific genes identified within genome sequencing projects in terms of their essentiality for bacterial replication. Based on this work, potent small molecule inhibitors on a selection of these targets have been identified and taken to preclinical and clinical development, resulting in mostly disappointing outcomes and discontinuation of the projects. Even though the non-optimal properties of the chemical libraries applied as a starting point has been identified as one contributing factor to the phenomenon, the reductionist target-based approach is considered to play a central role in this overall failure. The target-based approach suffers from two major limitations: the prerequisite for extensive target validation and the inability to reflect the complex interactions of biomolecules in a living organism.

Owing to these factors, phenotypic screening is regaining popularity in antibacterial drug discovery, as bacterial survival typically offers an easily detectable, biologically meaningful endpoint for the phenotypic assays. Regarding *C*. *pneumoniae*, technical limitations in the phenotypic growth assays exist due to the long life cycle and lack of general access to recombinant strains with fluorescent or luminescent reporter insertions. As the throughput capacity of the in vitro screening systems is limited, filtering chemical collections in silico is a plausible option.

The potential value of such a ligand-based virtual screening approach in finding new antichlamydial agents is demonstrated by our recent work describing a chemical space analysis of the previously identified plant-based antichlamydial compounds [25]. The chemometric analysis of 19 antichlamydial compounds showed them clustering together in a multidimensional chemical space defined by physicochemical descriptors such as size, aromaticity, polarity and lipophilicity. The identified privileged area within chemical space was utilized for a similarity screen on a small natural product library, allowing the selection of only 10% of the compounds for in vitro testing instead of the whole library, by selecting only the compounds closest to the identified antichlamydial hot spot. The in vitro *C*. *pneumoniae* growth inhibition assays revealed six new antichlamydial compounds, including mycophenolic acid, a clinically approved drug with known antibacterial activities. Of note, all six newly identified antichlamydial compounds are, despite sharing the same physicochemical properties, structurally different from each other and from the reference compounds. This finding indicates the power of this strategy in introducing more structural diversity to the lead molecules than any conventional structure-activity relationship model does. In addition, the described approach is generic in the sense that it is applicable to the lead discovery of also other microbes sharing limitations in in vitro screening capacity, given that a suitable reference set can be identified. Whether the identified privileged area in chemical space reflects a shared mechanism of action within the antichlamydial compounds or rather represents favourable physicochemical properties for penetrating into the chlamydial inclusion is a matter of further studies.

### 2.4. Pharmacophore-Based Design

Cationic antimicrobial peptides (AMPs) are a class of naturally occurring anti-infective agents essentially contributing to the innate immunity of all multicellular organisms [26]. The antimicrobial efficacy of most AMPs reflects their ability to disrupt bacterial membranes. Selective interaction of these peptides to microbial membranes is explained by a higher negatively charged outer surface and lack of cholesterol. As briefly mentioned above, most therapeutic challenges associated with *C. pneumoniae* infections are associated with the propensity to persistence, and finding therapies not relying on bacterial replication can be considered one of the ultimate goals of antichlamydial drug discovery. AMPs with inherent activity to the gram-negative outer membranes represent a means for also targeting *C. pneumoniae* in its nonreplicative form, and the potential for this approach has been illustrated by the ability of a cathelicidin-type AMP present in leukocytes to suppress *C. pneumoniae* infectivity when EBs are treated with the peptide [27].

Due to their nature as peptides, applicability of AMPs as therapeutic agents suffers from limitations in oral bioavailability and, more generally, inability to penetrate eukaryotic cell membranes. To overcome these challenges, a pharmacophore model for the synthesis of peptidomimetics has been applied. Instead of specific amino acid sequences, the antimicrobial activity of AMPs can be correlated with a given amount and topological distribution of charges, allowing the design of small molecules fulfilling these requirements. Applying such a strategy, a series of β^2,2^-amino acid derivatives has been synthesized and shown effective against both gram-negative and gram-positive bacteria [28]. These derivatives have been found to passively diffuse through phospholipid membranes and according to our recently published findings, two of them are able to disrupt the *C. pneumoniae* replication cycle even after the entry of the bacterium into epithelial cells [26]. Based on electron microscopy studies and targeted administration time studies, a dual mechanism of action was proposed, involving both destabilization of elementary bodies and targeting of the inclusion membrane in the early stages of infection. While the impact of the β^2,2^-amino acid derivatives on persistent *C. pneumoniae* infections is currently unexplored, these results represent the first report on AMP-derived compounds on intracellular bacteria and demonstrate the power of pharmacophore approach in the identification of innovative antichlamydial agents.

## 3. Plant Phenolics as Antichlamydial Agents

As mentioned above, the inhibitory effect of purified phenolic compounds on *C*. *pneumoniae* growth in epithelial cells was originally reported in 2006 [12]. A relatively large proportion of the assayed compounds were found to be highly active against *C. pneumoniae* (37% of the compounds showing >80% reduction in inclusion counts), subtle structural differences were found to result in remarkable differences in antichlamydial activity. An illustrative example on this is the difference in *C*. *pneumoniae* infective yield when treated with quercetin and its 7-methylated analogue rhamnetin, since rhamnetin completely blocks infective EB production at concentration 0.5 M, whereas only partial suppression in the infective yield is seen with quercetin treatment. The same phenomenon was later observed in a high-content screen for antichlamydial compounds among natural products, identifying biochanin A, a methylated isoflavone as a potent inhibitor of *C*. *pneumoniae* growth [29]. Similar to the differences between methylated rhamnetin and unmethylated quercetin studied by Alvesalo et al. [12], biochanin A was found to exert more potent antichlamydial effects than its unmethylated counterpart genistein.

Due to the general interest of dietary phenolics as health promoting agents, a remarkable number of studies have addressed the cellular processes affected by them. In Table 1, cellular processes affected by luteolin, a representative example of a dietary phenolic compound with high antichlamydial activity, are listed along with the impact of *C. pneumoniae* infection on the same processes. In the following chapters, these processes are discussed in view of their relevance to the antichlamydial effects excerted by luteolin. Excellent reviews on the signaling pathways targeted by luteolin can be found in the literature [30,31,32], and instead of aiming to reproduce a comprehensive presentation of all known aspects of luteolins biological activity, the current contribution highlights some aspects illustrating the multifaceted and often cell-type dependent modification of eukaryotic cells seen in both luteolin treatment and *C*. *pneumoniae* infection.

### 3.1. Oxidative Stress

As shown in Table 1, luteolin is known to counteract various pathological aspects reported within *C*. *pneumoniae* infection. It is not, however, clear to what extent these activities are related to the reduction of inclusion counts or infectious yield of the bacterium. In fact, in several cases it is not clear whether the reported *C. pneumoniae*-induced processes in the host cell are crucial for bacterial survival or rather represent innate host cell responses to the invading pathogen. As an example, *C*. *pneumoniae* infection has been reported to increase the levels of reactive oxygen species (ROS) in various cell types, including bronchial epithelium, platelets and macrophages [33,34]. In macrophages, Ca^2+^ -dependent production of ROS and nitric oxide (NO) is linked to persistence rather than active replication [35] and currently, there is no evidence on the role of ROS in promoting *C*. *pneumoniae* growth in epithelial cells. In addition, ROS scavenging agents ascorbic acid and N-acetylcysteine do not suppress *C. pneumoniae* growth in epithelial cells [16]. Based on these observations, it is likely that the inhibitory effects of luteolin and other related compounds on *C*. *pneumoniae* growth in epithelial cells is not directly linked to their ability to scavenge ROS but rather involve additional molecular targets.

Even though presumably not vital for chlamydial survival in most cell types, ROS play a central role in the induction of the pathological consequences of *C. pneumoniae* infection, such as cytokine and chemokine production and foam cell formation [34]. Owing to their antioxidative properties, flavonoids can thus be regarded as multifaceted antichlamydial agents, suppressing bacterial growth and alleviating the pathophysiological consequences of the infection. This concept is supported by the in vivo data on mice with acute *C*. *pneumoniae* infection. In this model, treatment with luteolin significantly decreased the bacterial load in the lungs and antibody titers in the bloodstream, but also diminished the inflammatory changes in histological samples of the lung [13]. Similar results have also been obtained with a phenol-rich extract of corn mint [11].

### 3.2. Mitogen-Activated Protein Kinases

In *C*. *pneumoniae* infections, induction of mitogen-activated protein kinases (MAPK) p38, extracellular signal regulated kinase ERK1/2 and c-Jun N-terminal kinase JNK is a well-known consequence of the infection in various cell types [36,37,38]. Activation of these ubiquitous stress-related signaling proteins is generally considered an essential step in the promotion of a proinflammatory and often proliferative phenotype. In the context of *C*. *pneumoniae* infection, p38, ERK1/2 and JNK have been reported to participate in different manifestations of the bacterium-induced phenotype changes of the host cell, such as cytokine, chemokine and adhesion molecule expression [38,39,40], foam cell formation [41] and increased proliferation [42,43]. Regarding *C. pneumoniae* entry, ERK1/2 activation has been reported to be crucial for successful internalization of EBs, presumably participating in the reorganization of actin microfilaments [44,45]. In these studies, *C*. *pneumoniae* entry to epithelial cells was prevented by a small molecule inhibitor of ERK1/2 activation by its upstream activator MEK.

Luteolin, as well as several other flavonoids, are also known to suppress p38, ERK1/2 and JNK activation in various cell types, including epithelial cells. This feature may, in part, explain the ability of luteolin-treated epithelial cells to resist *C*. *pneumoniae* infection even in an experimental setup in which the compounds withdrawn from culture medium prior to *C. pneumoniae* inoculation [12]. However, luteolin is highly active in reducing *C. pneumoniae* inclusion counts (MIC 9 µM) also when administered to the infected cells 2 h post inoculation, a time point in which EB internalization has already occurred. In the light of the current knowledge, it is not clear whether the inhibitory effect of luteolin on MAP kinase activation is directly related to its antichlamydial properties. Several above mentioned studies have demonstrated that small molecule inhibitors or genetic knockdown of the MAP kinase pathways suppress *C. pneumoniae*-induced pathophysiological changes. However, the impact of suppressing this signaling pathway on *C*. *pneumoniae* growth and survival in epithelial or other cell types has remained poorly understood. With a related pathogen *C*. *trachomatis*, activation of ERK1/2 has been reported to mediate the acquisition of host cell glycerophosholipids into chlamydial inclusion via activation of cytosolic phospholipase A_2_ (cPLA_2_) [46]. In this study, impairment of chlamydial growth was observed by blocking the cPLA_2_ activity, indicating that the pathway is essential for *C. trachomatis* in lipid acquisition. Despite the relatively close phylogenetic relationship of *C*. *trachomatis* and *C*. *pneumoniae*, differences in their ability to manipulate host cell signaling are known to exist and one study has reported even an increase in *C*. *pneumoniae* inclusion counts upon MEK/ERK1/2 inhibition [37].

**Figure 2:**
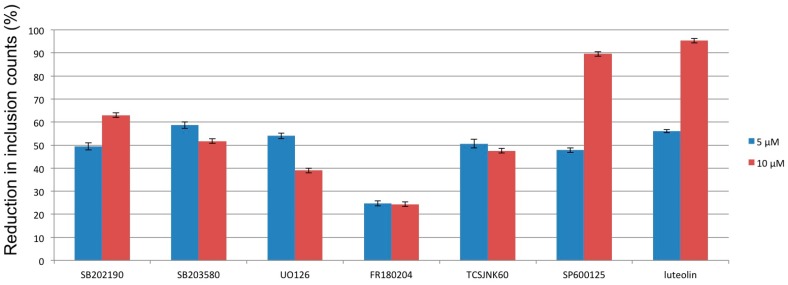
Impact of known MAP kinase inhibitors and luteolin on *C. pneumoniae* inclusion counts in human epithelial cells (HL). HL cells (400.000 cells/well) were inoculated with *C. pneumoniae* strain K7 (multiplicity of infection 0.2) and p38 (SB202190 and SB203580), ERK1/2 (UO126 and FR180204) and JNK (TCSJNK60 and SP60012) inhibitors were administered into the cultures 2 h post inoculation (p.i.), followed by immunofluorescent detection of chlamydial inclusions at 72 h p.i. All presented inhibition values are statistically significant compared to untreated control infections.

In order to understand the role of MAP kinase signaling in *C. pneumoniae* growth in respiratory epithelial cells, we have assayed commonly used p38, ERK1/2 and JNK inhibitors for their ability to reduce *C*. *pneumoniae* inclusion counts (Figure 2). In this assay, the small molecule inhibitors were added to the HL culture medium 2 h post inoculation in order to exclude chlamydial entry inhibition. According to these results, the ERK1/2 pathway-targeting probes UO126 and FR180204 did not have a remarkable effect on *C*. *pneumoniae* inclusion counts, while p38 inhibitors SB202190 and SB203580, as well as JNK inhibitors TCSJNK60 and SP600125, were more effective in blocking *C. pneumoniae* growth (unpublished data). These results indicate that especially p38 and JNK activation do seem to have a role not only in *C. pneumoniae*-induced pathophysiological processes but also in the intracellular survival of *C*. *pneumoniae*. The observation that none of these highly potent and selective probes was able to completely prevent inclusion formation indicates, however, that chlamydial survival strategies involve alternative and overlapping means for inducing a suitable environment. Considering the antichlamydial activities of luteolin and other plant phenolics, suppression of MAP kinase-driven signaling cascades seems one possible mechanism mediating these effects.

### 3.3. Intracellular Calcium Levels

Yet another cellular process, known to be affected by luteolin and other flavonoids and also associated with *C*. *pneumoniae* infection is the intracellular calcium homeostasis. In the context of *C. pneumoniae* entry into endothelial cells, genes encoding for components of Ca^2+^ trafficking system have been noted to activate within minutes of the cellular contact with the bacterium [47], and sustained, L-type calcium channel dependent calcium ion influx has been observed in *C. pneumoniae*–infected macrophages [35]. In macrophages, the elevated Ca^2+^ level mediates the ROS and NO production essential for the defence against pathogenic organisms, but from *C. pneumoniae* point of view, results in the persistent phenotype [48]. The studies on calcium channel blockers indicate that their administration to *C*. *pneumoniae*-infected macrophages rescue the infection from persistence and results in a more productive infection, and similarly, our observations on applying nonspecific (verapamil) or L-type specific (isradipin) calcium channel blockers to infected epithelial cells support the view that these compounds activate rather than suppress the acute infection [11]. The impact of phenolic compounds on L-type channel mediated Ca^2+^ influx varies depending on compound structure [49,50]. Of note, the phenolic compounds identified as the most potent antichlamydial compounds in the epithelial cell model [12] do not cluster together in this respect, as luteolin and octyl gallate, for instance, suppress Ca^2+^ influx while quercetin and morin stimulate it. Based on these findings, effects on cellular calcium homeostasis are not likely associated with the suppression of *C. pneumoniae* growth by phenolic compounds in epithelial cells but may, to some extent, be linked to the differential in vivo antichlamydial activity of these compounds [13].

Besides other biological processes, the calcium modulating effects of luteolin and other flavonoids have been linked to their ability to either induce or inhibit apoptosis in a cell type-dependent manner. In endothelial cells, luteolin blocks Ca^2+^ influx upon pathogenic stimuli, protecting these cells from apoptosis [51], whereas Ca influx into adipocytes is enhanced by luteolin treatment, leading to mitochondria apoptotic mediator release [52]. The antiapoptotic and proapoptotic signaling affected by luteolin also extends to various other signaling cascades, interconnecting with also those linked to *C. pneumoniae* infection, such as phosphatidylinsitide-3-kinase (PI3K) pathway [53,54] and Bcl-2 family proteins [55,56]. Similar to luteolin, the *C. pneumonia*-related apoptosis modulation seems to be cell type dependent, as for instance, epithelial cells turn resistant to apoptotic signals upon *C. pneumoniae* infection [57], while endothelial cells are shifted towards increased apoptotic tendency upon the infection [58]. The significance of these cell-type dependent effects on shared pathways for the in vitro or in vivo antichlamydial effects of luteolin remains unknown. Collectively, these features (e.g., Table 1) illustrate the need for more holistic approaches in future discovery and characterization of antichlamydial compounds.

## 4. Models of Persistent Infection

Mostly reflecting the early phases on *C*. *pneumoniae* research involving the isolation and characterization of bacterial strains from clinical samples, epithelial cells permissive to *C. pneumoniae* have been the most widely applied host systems on this pathogen. It is, however, well recognized that the acute infection model facilitated by centrifugation of inoculated cell monolayers does not fully reflect the in vivo infection by *C. pneumoniae*, and persistence models are thus necessary for comprehensive evaluation of any therapeutic strategies. Classically, cells of the monocyte–macrophage lineage have been considered the main reservoir of resident persistent forms of the bacterium [59], and these cells are thus often applied to study the spontaneous persistent infection in vitro [60]. However, the chlamydial persistence is not limited to cells of the immune system, as the chronic infection can be induced also in various other cell types, such as fibroblasts, smooth muscle and endothelial cells. Different means for the induction of persistence in these cell types can be applied, such as iron or other nutrient depletion or treatment with penicillin or interferon gamma. Recent studies on the bacterial and host cell gene expression profiles indicate that the biological state of the infection differs depending on the means used for persistence induction, and it is currently not clear which of the models best represents the in vivo infection [61,62,63]. Besides the external inducers, a degree of persistent state can also be triggered by a continuous infection models in epithelial cells, representing a mixed phenotype with a proportion of the bacterial population being in an antibiotic-refractory state [64,65]. Collectively, these models provide the means for in vitro characterization of antichlamydial compounds on different aspects of persistence.

## 5. Conclusions

Faced by the poor success rate during previous decades, antimicrobial research is struggling to find new approaches and reevaluating the old approaches to develop effective antibiotics. The examples presented in this review show that despite the challenging features of *C*. *pneumoniae* as a drug target, a spectrum of strategies are applicable for identifying nonconventional inhibitors of this hard-to-treat pathogen.

Acknowledging the complexity of disease processes and living organisms in general, a shift from a “single drug, single target” concept toward attempts to affect whole biological networks is been arising within biomolecular screening [66], and increased understanding on the pharmacology of classical antibiotics also implies that their in vivo efficacy does not rely only on limiting bacterial replication, but also on immunomodulatory and potentially other activities [67]. Combination treatments and multifaceted therapy approaches can thus be expected to increase their popularity within antibacterials and other therapy areas, and *C. pneumoniae* cannot be expected to be an exception to this phenomenon. As illustrated by the findings on multiple interconnected and partially overlapping pathways involved in *C. pneumoniae* entering epithelial cells [47] and the partial impact of inhibiting different MAP kinases on *C. pneumoniae* growth presented in this contribution, *C. pneumoniae* likely is seldom dependent on only one pathway or signaling cascade for its survival. Covering the complementary, alternative and overlapping *C. pneumoniae* survival strategies can thus be considered vital for successful antichlamydial agents. The various biological activities exerted by dietary phenolics make them an attractive research topic as first generation nonconventional antichlamydial agents, but future studies are necessary to evaluate the relevance of individual pathways in the context of *C. pneumoniae* infection.

## Figures and Tables

**Table 1:** Cellular processes affected by *Chlamydia pneumoniae* and luteolin, a representative example of plant phenolics suppressing *C. pneumoniae* growth.

Target	*C. pneumoniae*	Luteolin	References
ROS	+	−	[30,33,34]
NF-κB	+	−	[31,39]
MAP kinases	+	+/−	[32,36]
PI3 kinase	+	−	[53,54]
Cytosolic Ca^2+^	+	+/−	[35,36,37,38,39,40,41,42,43,44,45,46,47,48,49,50,51,52]
Bcl-2 proteins	−	+/−	[56,58]

“+” indicates an inducing / stimulating effect and “−“ indicates a suppressing/inhibitory effect. “+/−“ indicates that the reported effect is cell type dependent, typically with opposing effects in malignant and nonmalignant cells. ROS = reactive oxygen species, NF-κB = nuclear factor kappaB, MAP kinase = mitogen-activated protein kinase, PI3 kinase = phosphatidylinositide-3-kinase.
